# The effect of varus knee deformities on the ankle alignment in patients with knee osteoarthritis

**DOI:** 10.1186/s13018-019-1191-0

**Published:** 2019-05-15

**Authors:** Kai Xie, Xuequan Han, Xu Jiang, Songtao Ai, Kerong Dai, Zhifeng Yu, Haishan Wu, Xinhua Qu, Mengning Yan

**Affiliations:** 1 Shanghai Key Laboratory of Orthopaedic Implants, Department of Orthopaedic Surgery, Shanghai Ninth People’s Hospital, Shanghai Jiao Tong University School of Medicine, 639 Zhizaoju Road, Shanghai, China; 2 Department of Radiology, Shanghai Ninth People’s Hospital, Shanghai Jiao Tong University School of Medicine, 639 Zhizaoju Road, Shanghai, China; 3 Department of Bone and Joint Surgery, Renji Hospital, School of Medicine, Shanghai Jiao Tong University School of Medicine, 145 Middle Shandong Road, Shanghai, China

**Keywords:** Total knee arthroplasty, Knee alignment, Ankle malalignment, Varus knee deformity

## Abstract

**Background:**

We evaluated the compensatory change in ankle alignment due to knee malalignment and its relationship with varus knee deformities, as well as sex differences in compensation.

**Methods:**

From October 2016 to September 2017, 103 patients with end-stage knee osteoarthritis underwent primary total knee arthroplasty (TKA). Ninety-five knees (78 patients) were included. The hip-knee-ankle angle (HKA) and ankle alignment and tilt were evaluated with full-leg standing anteroposterior radiographs. The ankle alignment was estimated according to the tibiotalar angle, tibial anterior surface angle (TAS), and lateral distal tibial angle. The talar tilt angle (TT), anatomical talocrural angle, angle between the tibial plateau and distal tibial plafond, angles between the ground and distal tibial plafond, and angles between the ground and upper talus were measured to evaluate ankle tilt. The patients were separated into two sex-based groups; correlations between the HKA and ankle parameters were estimated.

**Results:**

The mean HKA in men and women was 8.16 ± 4.36° and 7.69 ± 5.93°, respectively. The relative tilt of the talus and distal tibia plafond to the ground was increased when varus knee deformities progressed. In women, there was a positive correlation between the knee alignment and TAS (*r* = − 0.295, *p* = 0.016). As the knee mechanical axis became more varus, the distal tibia plafond became more valgus. In women, a negative correlation was observed between the HKA and TT (*r* = − 0.359, *p* = 0.003). Compensatory changes in the ankle alignment and TT to knee alignment were not observed in men.

**Conclusion:**

Compensatory ankle changes should be considered before TKA.

## Background

Osteoarthritis (OA) is a major chronic disease worldwide, affecting more than 10% of people over 60 years old with joint pain and disability [1]. The knee is a complex joint that bears a high load during activities of daily life. Inappropriate joint biomechanics are a main risk factor of knee OA [2]. The joint biomechanics are directly affected by lower extremity malalignment caused by an anatomical deformity. The mechanical alignment of the lower extremity is highly determined by the morphology of the hip, knee, and ankle joints. Varus deformities of the knee cause overloading and cartilage wear in the medial compartment, which could exacerbate degeneration of the knee joint. Severe knee OA is a long-term pathological process that could change the alignment of the entire lower limb and accelerate degeneration of the ankle joint [3].

Recently, ankle OA has received more attention from orthopedists than it has in the past; however, the prevalence of symptomatic ankle OA is still unknown. Trauma is the most prevalent etiology of ankle OA, and posttraumatic OA accounts for approximately 70% of ankle OA cases [4–6]. The prevalence of primary ankle OA is rare, whereas 35.2% of patients with knee OA have a radiologic degenerative change in the ankle before undergoing total knee arthroplasty (TKA) [7]. The ankle would respond to varus knee deformities as an important part of lower extremity alignment. A few previous studies have reported that radiologic malalignment occurs in both the ankle and hindfoot joints secondary to varus knee deformities [7–9]. Valgus ankle alignment and a lateral metaphyseal, collapsed, distal tibia were observed in patients with varus knee deformities. For hindfoot alignment, an increase in varus knee deformities would make the hindfoot alignment more valgus. However, no evidence supports the idea that compensatory changes in the ankle alignment would increase with the aggravation of varus knee deformities. It is unclear whether compensatory changes in the ankle alignment differ based on sex. The objectives of the current retrospective study were to estimate the change in ankle alignment to compensate for varus knee deformities and evaluate differences in sex regarding these compensatory changes.

## Methods

### Patient enrollment

The current study was approved by our institutional review board (SH9H-2018-179-T137). Informed consent was obtained from all individual participants included in the study. Between October 2016 and September 2017, 103 patients with end-stage knee OA underwent primary TKA at our center. All patients underwent full-leg standing anteroposterior (AP) radiography before TKA. The inclusion criteria were TKA patients with radiologically seen knee OA and joint pain and/or dysfunction. The exclusion criteria were inadequate or missing AP radiographs, history of ipsilateral lower extremity surgery before TKA, and valgus knee alignment before TKA. Twenty-five patients were excluded (Table 1).

**Table 1:** Exclusions from the whole cohort

Reason for exclusion	Number of patients
Inadequate AP radiograph	3
Missing AP radiograph	5
History of ipsilateral lower extremity surgery before TKA	4
Valgus knee alignment before TKA	13
Total	25

Ninety-five knees in 78 patients were enrolled. Twenty-four patients (29 knees) were men, and 54 (66 knees) were women. The mean age of the patients was 68.7 years (range 53–85). Seventeen patients underwent bilateral TKA, 39 patients underwent right-side TKA, and 22 patients underwent left-side TKA. The knee and ankle on the surgical side were evaluated in the following radiographic assessments.

### Radiographic assessment

**Fig. 1:**
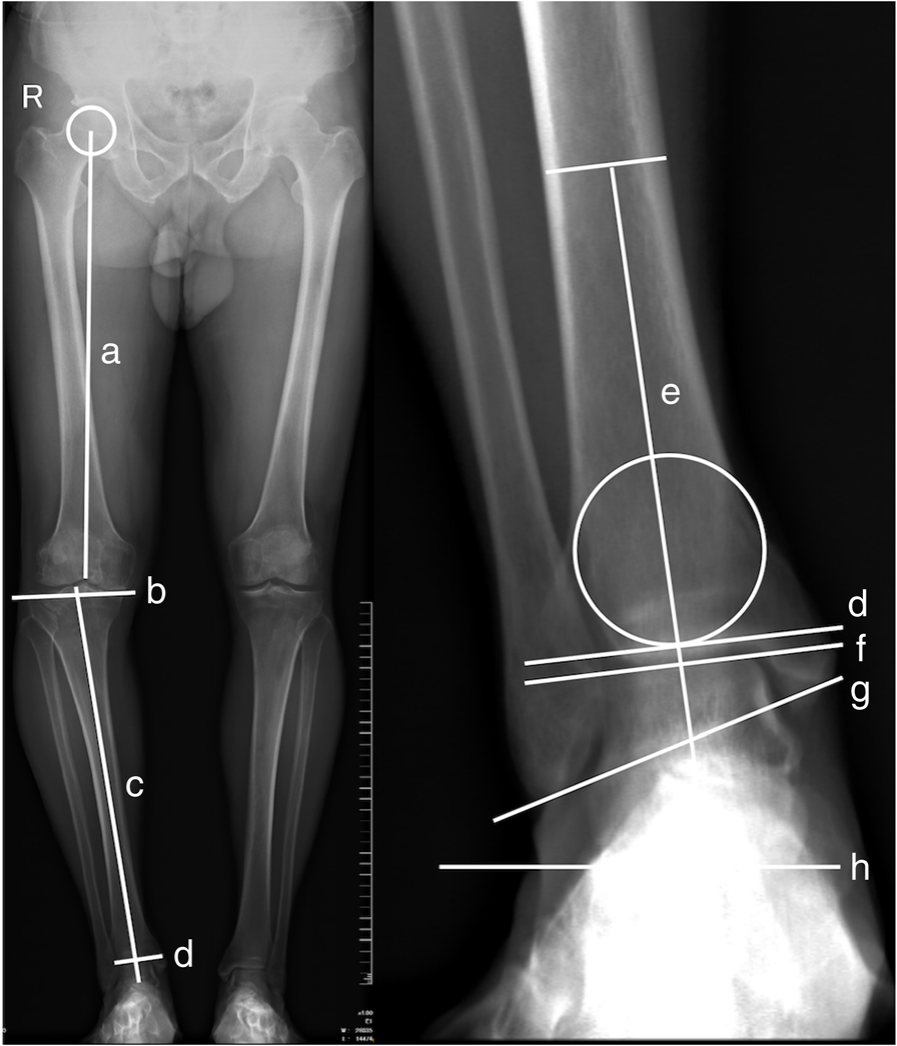
Measurement of HKA and ankle parameters based on full-leg standing anteroposterior radiographs. TTA was defined as the medial angle between “e” and “f.” TAS was defined as the medial angle between “e” and “d.” LDTA was defined as the lateral angle between “c” and “d.” TT was defined as the angle between “d” and “f.” GT was defined as the angles between “f” and “h.” GP was defined as the angle between “d” and “h.” PP was defined as the angles between “b” and “d.” TC was defined as the medial angles between “e” and “g.” Line a was the line from the center of the femoral head to the center of the knee. Line b was the joint surface of the tibial plateau. Line c was the line from the center of the tibial plateau to the center of ankle. Line d was the distal tibial plafond. Line e was the anatomical axis of the tibia. Line f was the upper joint surface of the talus. Line g was the line from medial malleolus to lateral malleolus. Line h was the ground surface

Patients underwent full-leg standing digital AP radiographs with the patellae forward and in full knee extension to avoid measuring errors caused by lower extremity rotation as previously described [10]. Digital radiographs were acquired using Kodak DirectView (Kodak, Rochester, NY, USA) from all patients before they underwent TKA, and stored in the General Electric system (General Electric, Boston, MA, USA). The level of knee OA was rated according to the Kellgren-Lawrence radiographic classification of OA [11, 12]. Materialise Mimics version 20.0 (Materialise, Leuven, Belgium) was used to perform all measurements. The hip-knee-ankle angle (HKA), tibiotalar angle (TTA), tibial anterior surface angle (TAS), lateral distal tibial angle (LDTA), talar tilt angle (TT), anatomical talocrural angle (TC), angle between the tibial plateau and distal tibial plafond (PP), angles between the ground surface and distal tibial plafond (GP), and angles between the ground surface and upper talus (GT) were measured using full-leg standing digital AP radiographs (Fig. 1).

Varus knee deformities were evaluated with the HKA, while tibiotalar joint alignment was evaluated with the TTA, as previously reported [4, 8]. The femur mechanical axis was defined as a line from the center of the femoral head to the center of the knee. The tibia mechanical axis was defined as a line from the center of the tibial plateau to the center of the ankle. The HKA was defined as the angle between the mechanical axes of the femur and tibia. A HKA greater than or equal to 3° was defined as varus knee deformity [13]. The TTA was defined as the angle between the tibia anatomical axis and talus upper surface.

The TAS and LDTA were used to evaluate compensatory changes in the distal tibial plafond. The TAS was defined as the angle between the tibia anatomical axis and distal tibial plafond. The LDTA was defined as the lateral angle between the tibia mechanical axis and distal tibial plafond. The tilt of the talus relative to the distal tibial plafond and ground was evaluated with the TT and GT. The TT was defined as the angle between the distal tibia plafond and the upper joint surface of the talus. For the TT, an angle opening at the medial side was defined as a positive value. In the software that was used, a horizontal line indicated the ground surface. The GT was defined as the angle between the ground surface and upper talus, which was evaluated using the angle between a horizontal line and the upper talus. The TC was defined as the angle between the anatomical axis and a line from the medial malleolus to the lateral malleolus. The relative tilt of the talus upper joint surface to the tibial plateau and ground was evaluated with the PP and GP. The correlation between the HKA angle and ankle parameters was estimated.

### Statistical analysis

SPSS version 23.0 (IBM Corp., Armonk, NY, USA) was used to perform all statistical analyses. A *p* value less than 0.05 was considered statistically significant. The correlation between the HKA and other ankle parameters was evaluated with scatter plots and a two-tailed Pearson’s correlation test. A two-tailed *t* test was used to compare the HKA and ankle parameters in male and female patients.

## Results

According to the Kellgren-Lawrence radiographic classification of OA, 20 knees had grade 3 and 75 knees had grade 4. The mean HKA in grade 4 group was 9.05 ± 5.43°, which was significantly higher than that in the grade 3 group (3.26 ± 2.38°). As presented in Table 2, there were significant differences in ankle morphology (TAS, TT, GP, GT, and TC) between the grade 3 and grade 4 groups, indicating that varus knee deformity had a significant influence on ankle alignment in knee OA patients.

**Table 2:** Characteristics of patients with different grade of knee OA

Characteristic	Grade 3	Grade 4	*p*
Number of patients	18	60	/
Number of knees	20	75	/
Mean age (year)	68.10 ± 6.03	68.80 ± 6.26	0.439
Disease duration	6.60 ± 5.86	7.34 ± 4.36	0.363
HKA	3.26 ± 2.38	9.06 ± 5.43	< 0.001
TTA	88.86 ± 3.34	89.69 ± 3.09	0.296
TAS	87.06 ± 3.53	88.70 ± 3.07	0.043
LDTA	92.97 ± 3.39	91.57 ± 2.82	0.062
TT	1.97 ± 0.92	1.24 ± 1.55	0.047
Ground-plafond (GP)	7.37 ± 3.42	9.28 ± 3.84	0.046
Ground-talus (GT)	5.94 ± 3.10	8.12 ± 4.24	0.033
Plateau-plafond (PP)	7.11 ± 4.08	6.49 ± 3.75	0.514
TC	75.20 ± 4.66	77.48 ± 3.40	0.016

**Fig. 2:**
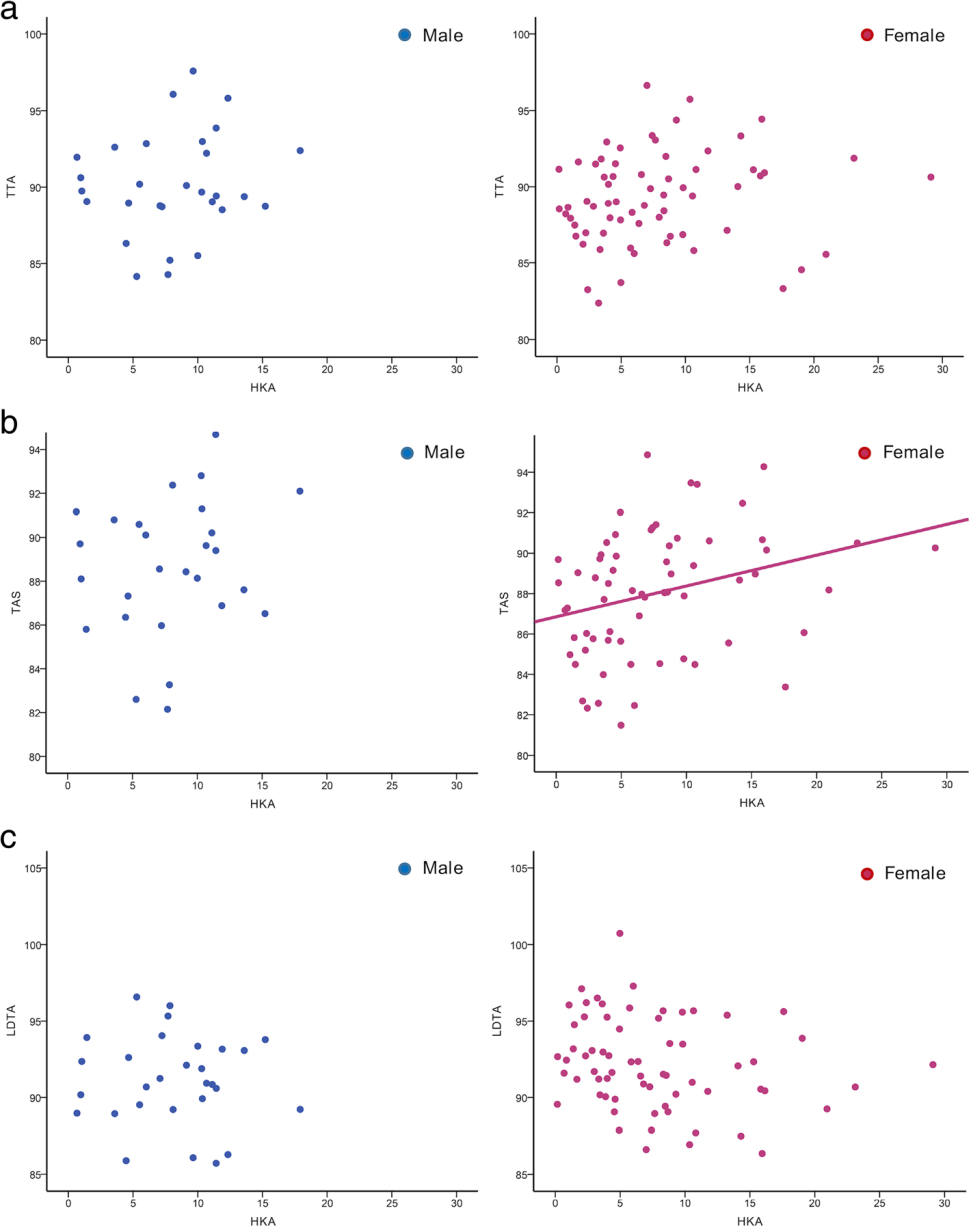
The correlation analysis between HKA and compensatory ankle change (**a** TTA, **b** TAS, and **c** LDTA.The scatter diagram indicated a correlation between the HKA and TAS in female group (**b**)

There was no statistically significant difference in the mean HKA and ankle parameters between men and women (Table 3). The correlation between the HKA and ankle parameters is presented in Table 4. The mean HKA in men and women was 8.16 ± 4.36° and 7.69 ± 5.93°, respectively. The mean TTA in men and women were 89.84 ± 3.35° and 90.77 ± 3.03°, respectively. The varus-to-normal tibiotalar joint alignment was observed in patients in both groups, and no statistically significant correlation was observed between the knee alignment and TTA. However, a significant correlation was observed between the knee alignment and TAS in women. In these patients, as the knee mechanical axis became more varus, the distal tibia plafond became more valgus (*r* = 0.295, *p* = 0.016). The mean TAS in men and women was 89.10 ± 3.49° and 88.03 ± 3.07°, respectively. The lateral metaphyseal collapse of the distal tibia plafond was observed based on the measurement of the LDTA. The mean LDTAs in men and women were 91.11 ± 2.93° and 92.19 ± 2.97°, respectively. In women, although statistically insignificant, the LDTA decreased when the knee alignment became varus (*r* = − 0.219, *p* = 0.077) (Fig. 2).

**Table 3:** Characteristics of patients

Characteristic	Male	Female	*p*
Number of patients	24	54	/
Number of knees	29	66	/
Right/left	18/11	38/28	/
Mean age (year)	69.83 ± 6.329	68.17 ± 6.25	0.124
HKA	8.16 ± 4.36	7.69 ± 5.93	0.439
TTA	89.84 ± 3.35	90.77 ± 3.03	0.363
TAS	89.10 ± 3.49	88.03 ± 3.07	0.702
LDTA	91.11 ± 2.93	92.19 ± 2.97	0.186
TT	1.34 ± 1.57	1.42 ± 1.43	0.136
Ground-plafond (GP)	8.44 ± 3.72	9.07 ± 3.88	0.107
Ground-talus (GT)	7.19 ± 4.06	7.87 ± 4.14	0.817
Plateau-plafond (PP)	6.12 ± 3.34	6.84 ± 4.00	0.463
TC	77.43 ± 3.76	76.82 ± 3.82	0.460

*HKA* hip-knee-ankle angle, *TTA* tibiotalar angle, *TAS* tibial anterior surface angle, *LDTA* lateral distal tibial angle, *TT* talar tilt angle, *TC* anatomical talocrural angle, *PP* angle between the tibial plateau and distal tibial plafond, *GP* angle between the ground surface and distal tibial plafond, *GT* angle between the ground surface and upper talus

**Table 4:** The correlation of HKA with ankle parameters in male and female group

Ankle parameter	Correlation	*p*
Male		
TTA	0.154	0.425
TAS	0.246	0.199
LDTA	− 0.065	0.739
TT	− 0.177	0.360
Ground-plafond (GP)	0.455	0.013
Ground-talus (GT)	0.476	0.009
Plateau-plafond (PP)	0.191	0.321
TC	− 0.001	0.994
Female		
TTA	0.130	0.297
TAS	0.295	0.016
LDTA	− 0.219	0.077
TT	− 0.359	0.003
Ground-plafond (GP)	0.683	< 0.001
Ground-talus (GT)	0.751	< 0.001
Plateau-plafond (PP)	0.331	0.007
TC	0.203	0.103

*HKA* hip-knee-ankle angle, *TTA* tibiotalar angle, *TAS* tibial anterior surface angle, *LDTA* lateral distal tibial angle, *TT* talar tilt angle, *TC* anatomical talocrural angle, *PP* angle between the tibial plateau and distal tibial plafond, *GP* angle between the ground surface and distal tibial plafond, *GT* angle between the ground surface and upper talus

**Fig. 3:**
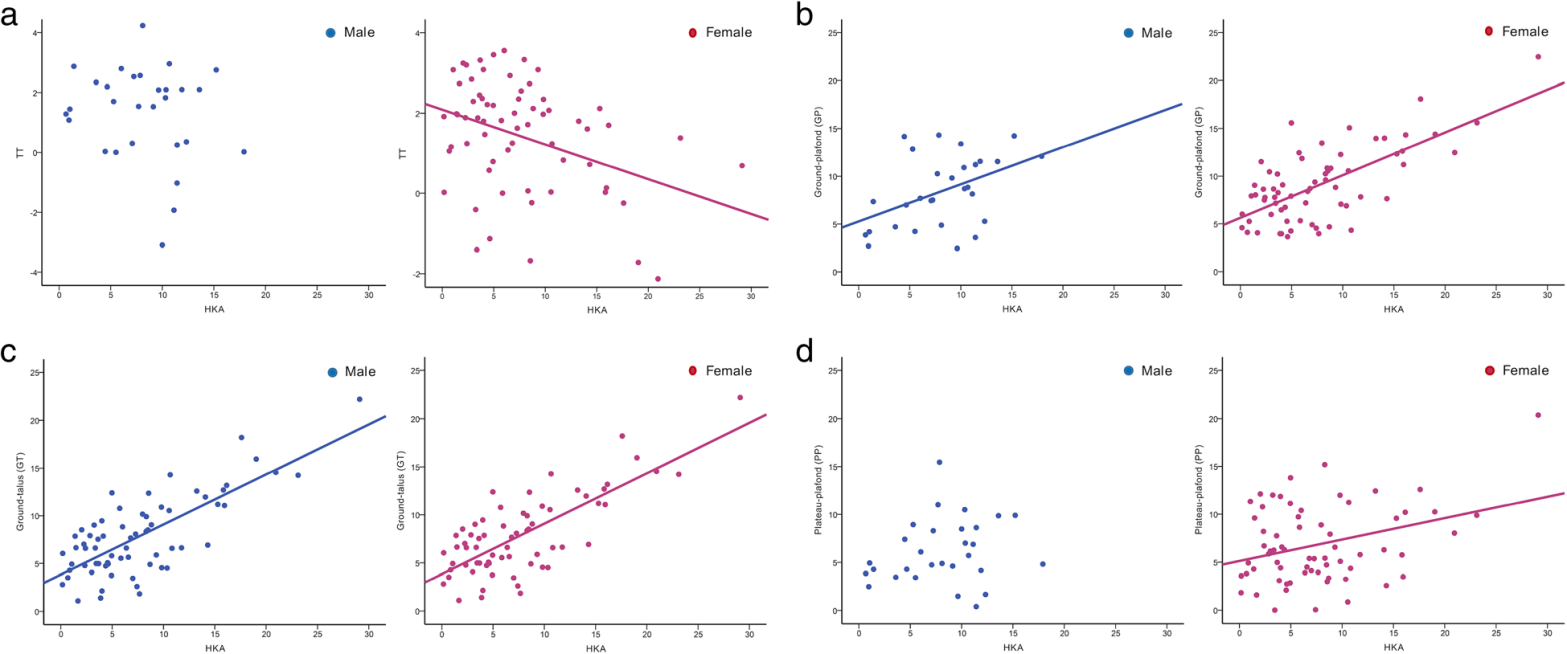
The correlation analysis between the HKA and tilt of ankle (**a** TT, **b** GP, **c** GT, **d** PP) in both male and female patients

Compensatory change in the ankle tilt was observed in both men and women (Fig. 3). The TT in men and women was 1.34 ± 1.57° and 1.42 ± 1.43°, respectively. A negative correlation was observed between the HKA and TT (*r* = − 0.359, *p* = 0.003), confirming that there were compensatory changes in the ankle to a varus knee deformity. However, there was no correlation between the knee alignment and TT in men. The mean GT in men and women were 7.19 ± 4.06° and 7.87 ± 4.14°, respectively. The mean GP in men and women were 8.44 ± 3.72° and 9.07 ± 3.88°, respectively. Varus knee deformities had a significant influence on the GT (*r* = 0.751, *p* < 0.001) and GP (*r* = 0.683, *p* < 0.001) angles in women. In men, the HKA was moderately and positively correlated with the GT (*r* = 0.455, *p* = 0.009) and GP (*r* = 0.455, *p* < 0.013). In women, because the lateral metaphyses of the distal tibia plafond and tibial plateau collapsed, the angle between the tibial plateau and distal tibial plafond was also increased as the knee became more varus (*r* = 0.331, *p* = 0.007). The mean TC in men and women was 77.43 ± 3.76° and 76.82 ± 3.82°, respectively. There was no correlation between the TC and HKA.

## Discussion

The objectives of the current retrospective study were to estimate the change in ankle alignment to compensate for varus knee deformities and evaluate differences in sex regarding these compensatory changes. In women, the ankle alignment was significantly influenced by varus knee deformities, and as the knee mechanical axis became more varus, the distal tibia plafond secondarily became more valgus. However, a correlation between changes in the ankle alignment and knee mechanical axis was not found in male patients. Moreover, we found that abnormal biomechanics in the ankle were significantly associated with varus knee deformities in both men and women.

Although the effect of varus knee deformities on degenerative changes in the ankle has not been fully established, the relationship between varus knee deformities and ankle OA has been proven [14]. The most prevalent type of ankle OA is posttraumatic OA, while the prevalence of primary and secondary ankle OA is rarely reported. However, as previously described, the prevalence of ankle OA ranges from 28.8 to 35.2% in TKA patients without a history of ankle fracture [7, 15]. In a study on cadaveric donors, severe ankle degeneration always existed along with knee degeneration [16].

Degeneration of the lower limb joints is highly related to the joint alignment. In the current study, the LDTA decreased as the knee alignment became more varus, indicating lateral metaphyseal collapse and valgus change in the distal tibia plafond. Our results show that compensatory changes in the ankle to varus knee deformities mainly occur at the distal tibial side, which could influence the ankle tilt and cause an abnormal biomechanical state in the tibiotalar joint. Therefore, we believe that valgus change in the distal tibial plafond and increased ankle tilt could be the main causes of ankle degeneration due to varus knee deformities.

Another interesting finding of the current study is that compensatory change in the ankle tilt to varus knee deformities could increase abnormal forces on the ankle. In women, the TT decreased as the knee alignment became more varus, indicating lateral tilt of the tibiotalar joint. In addition, the relative tilt of the talus and distal tibia plafond to the ground also increased as varus knee deformities progressed. Increased tilt of the talus and distal tibia plafond could change the biomechanical state of the ankle, which could lead to narrowing of the medial ankle joint space and collapse of the lateral metaphysis of the distal tibia plafond. It is still uncertain whether the compensatory change in ankle morphology could recover after TKA. Therefore, it is necessary to evaluate the ankle alignment in knee OA patients preoperatively and postoperatively, which could further confirm the effect of varus knee deformity on the ankle alignment.

A more perplexing area of uncertainty is whether the compensatory change in ankle morphology negatively affects the outcome of TKA. TKA is the most cost-effective method to treat end-stage knee degeneration [17]. In past decades, the surgical techniques, perioperative management, and prosthesis survivorship of TKA have improved dramatically, but the patient-reported dissatisfaction rate of TKA ranges from 11 to 25% [18, 19]. Many factors could affect the patient’s satisfaction with TKA, such as age, preoperative mental health, and postoperative knee stability [20, 21]. In recent years, the effects of ankle and foot stability on TKA outcomes have received more attention. Gursu et al. [9] evaluated the ankle morphology and alignment in 80 knees with at least 10° of varus deformities and concluded that overcorrecting the tilt of the distal tibia plafond and ankle alignment could be the main reason for postoperative ankle pain. As mentioned before, in women, lateral tilt of the distal tibia plafond and valgus changes to it would increase when the knee alignment becomes more varus. In such patients, leaving a residual varus knee deformity may lead to a better clinical outcome, which corresponds to our clinical experience. For most arthroplasty surgeons, it is uncommon to estimate the alignment and abnormal biomechanical state of the ankle before TKA. Based on the results of the current study and previous articles, we suggest performing a careful preoperative examination for ankle deformities in patients with knee OA, especially women with large varus knee deformities.

The current study has some limitations. First, the number of men was not large due to the relatively low prevalence of TKA in the male population. To our knowledge, we are the first to report sex differences in terms of compensatory change in the ankle alignment due to varus knee deformities. Moreover, the findings of the current study could provide a new perspective for further research. Second, the anatomic data that were used were from the Asian population, and the results of this study may not be generalized to people of other ethnicities. Third, we did not investigate the influence of hand dominance and patient complaints (such as joint pain and range of motion) on compensatory change in ankle alignment due to knee malalignment.

## Conclusions

We observed a significant correlation between ankle malalignment and varus knee deformity in patients with knee OA. Compensatory changes in the ankle should be considered before TKA is performed. More large-scale and well-designed research studies are needed to fully demonstrate the effect of knee malalignment on ipsilateral and contralateral ankle alignment.

## Abbreviations

AP: Anteroposterior
GP: Angle between the ground surface and distal tibial plafond
GT: Angle between the ground surface and upper talus
HKA: Hip-knee-ankle angle
LDTA: Lateral distal tibial angle
OA: Osteoarthritis
PP: Angle between the tibial plateau and distal tibial plafond
TAS: Tibial anterior surface angle
TC: Anatomical talocrural angle
TKA: Total knee arthroplasty
TT: Talar tilt angle
TTA: Tibiotalar angle
